# Personal Workplace Relationships: Unifying an Understudied Area of Organizational and Personal Life

**DOI:** 10.3390/bs13090760

**Published:** 2023-09-13

**Authors:** Rebecca M. Chory, Sean M. Horan

**Affiliations:** 1 Department of Management, Frostburg State University, Frostburg, MD 21532, USA; 2 Department of Communication, Fairfield University, Fairfield, CT 06824, USA

## Abstract

Relationships that cross the work–life domain have long been of interest to scholars in multiple disciplines, including Communication, Management, and Psychology. Close relationships that span work–life borders are called *personal workplace relationships*. Personal workplace relationships are voluntary informal relationships between two members of the same organization. These relationships are mutual and consensual and have a relatively strong emotional component. They involve the partners knowing and communicating with each other as unique individuals. The goal of this Special Issue (“Personal Workplace Relationships: Implications for Work and Life in a Rapidly Changing Society”) is to explore this specific form of work–life intersection. To that end, we present the scholarly work of researchers from diverse backgrounds who share the goal of better understanding workplace relationships. In this opening essay, we describe how we began to study this area, we preview the articles in this Special Issue, and we conclude with recommendations for future research on personal workplace relationships.

For decades, researchers across disciplines have studied work–life boundary management and work–life balance. We are no different in this regard. Chory, with her training in Organizational Communication and Industrial/Organizational Psychology, and Horan, with his work–life coursework from Patrice Buzzanell and Mary Hoffman, merged their interests in 2007 to begin a career-long collaboration examining work–life boundaries, blending, and management in the context of workplace romance. Throughout our careers, we have also taught this content, with Chory creating and teaching undergraduate Management and MBA “Sex, Gender, and Workplace Relationships” courses, as well as undergraduate and graduate courses in Leadership and Organizational Communication, and Horan through his undergraduate “Dark Side of Communication” course and his MA level “Work–Life Intersections”. 

Throughout our teaching, we have clearly stated that balance is a problematic metaphor—if we all attained work–life balance, we would spend 50% of our time in the work domain and 50% in the life domain. Instead, we believe that employees seek satisfaction with their distribution of time and effort in work and life. From the outside, someone’s life could be totally imbalanced by working 90 hours a week, but that person is satisfied because (s)he wants to work this much. Rather than seeking balance and studying that concept specifically, we instead examine how the work and life domains overlap (i.e., intersect) and the related spillover/blending. Similar to Horan’s graduate course, we use the terminology “work–life intersections” to describe how work and life overlap and how individuals cross domains, manage boundaries between the two domains, and deal with spillover/blending. In line with this interest and expertise, our Special Issue, “Personal Workplace Relationships: Implications for Work and Life in a Rapidly Changing Society”, explores a specific form of work–life intersection and spillover/blending: personal workplace relationships.

Personal workplace relationships are relationships between organizational members that span both the “work” and “life” domains and are marked by communication that crosses the work–life border [1,2]. These relationships involve both task (e.g., supervisors training new hires) and relational (e.g., coworkers discussing parenting challenges) communication. Formally, *personal workplace relationships* (PWRs) are defined as “voluntary, informal, mutual, and consensual relationships between two members of the same organization that are marked by a strong emotional component and the partners’ knowing and communicating with each other as whole, unique persons” [2] (p. 47). PWRs may take various forms, including friendships or romances that developed in the workplace or existed prior to employment in the shared organization; married couples, in laws, and family friends in family businesses; casual sexual relationships between co-workers; extramarital affairs; and “work spouses”. (Although popularized by the media, we find the academic treatment of “work spouses” less clear [2]; e.g., what distinguishes a work spouse from a best friend at work? Some work spouses have had sexual relations [3], what role does physical attraction play?)

To better understand the orientation of this Special Issue, please allow us to describe our backgrounds and the history of our collaboration on PWRs.

## 1. Background

We both hold BA, MA, and PhD degrees in Communication. As a PhD student at Michigan State University, Chory studied Organizational and Mass Communication, Management, and Industrial/Organizational Psychology. Over the last 20 years, she has attained tenured Full Professor status in both the Communication Studies and Management disciplines. Horan earned his PhD from West Virginia University, focusing primarily on communication in relationships. Horan earned tenured Full Professor status in Communication and, throughout his career, collaborated with Chory on research. Chory was Horan’s professor and a member of his dissertation committee.

In 2007, Chory taught an Advanced Organizational Communication course, in which second-semester doctoral student Horan was enrolled. Chory required all doctoral students in the course to design and execute an original empirical research project pertaining to the course content. Horan held interests in relational communication, which he applied in Chory’s course. Marrying related interests, we began studying interpersonal relationships that operated both within and outside of the workplace, beginning with workplace romances.

Since our initial workplace romance study, we have continued to research relationships that vary in closeness and cross work–life borders. We have collaborated on further studies of workplace romance [4,5,6,7] and developed the topic area through work with our graduate students [8,9,10,11] and colleagues [10,12,13,14]. Our collaborative workplace romance research continues at the time of this writing. Chory has also conducted research on workplace friendships [15], particularly cross-sex workplace friendships [16].

At the time our collaborative research began, we took note of several things. First, the research and theorizing on blended relationships, i.e., those that span work and personal lives, were underdeveloped. Despite the fact that such relationships are common and impact both internal and external stakeholders, the research was relatively sparse across disciplines. We were not the first to note this [17], yet it continues today.

Second, not only was the scholarly work on blended relationships underdeveloped but it tended to be siloed *across* disciplines. Early on, Management scholars focused on workplace romances. They primarily concentrated on formal organizational policies [18,19], managerial interventions [20,21,22,23,24], and the legal aspects of workplace romances, including their effects on sexual harassment [17,25,26,27,28]. Both Management and Communication researchers addressed gender and status dynamics in workplace romances [22,23,24,29,30,31,32], as well as third-party attributions of motives for workplace romance [33,34], sometimes in combination [23,24,35,36].

On the other hand, Communication and Psychology scholars were leading the way in the study of workplace friendships during this time (pre-2005). Communication scholars researched peer coworker communication and relationships [37,38,39], the development of workplace friendships [40], and the dialectical tensions experienced by workplace friends [41]. Likewise, in Psychology, workplace friendships and gender differences in PWRs [29,42,43,44] were early and popular topics.

Even *within* our home disciplines, the work was (and is) somewhat siloed [45]. Interpersonal and Organizational Communication researchers tend to focus on their own sub-areas of research, at times failing to integrate the perspectives of one another. In Management, scholars using a social network approach are predominant in the published research on workplace friendships [46,47,48,49], whereas human resources researchers continue to address workplace romance and sexual harassment policy development [50,51,52,53,54].

Third, PWR scholarship appeals to a broad audience due to its interdisciplinary nature. However, because the study of PWRs does not fit exclusively in one, or even two, disciplines, research on the topic is often left in the margins. Locating suitable peer-reviewed academic journals open to publishing such work can also be challenging. We applaud and remain grateful to the scholars who, early on, saw the importance of studying PWRs and persevered in getting their research published in high-quality journals. In particular, we acknowledge the efforts of Mainiero [19,21], Dillard [30,34,35], Powell [32,55], Pierce and Aguinis [25,26], Sias [39,40], Bridge and Baxter [41], and Quinn [24], to name a few.

Given our professional backgrounds and scholarly interests, our approach to research and theory building on PWRs has always been interdisciplinary. After all, how can one understand a workplace friendship or romance without relying on both Relational and Organizational Communication perspectives and Organizational Behavior, Business Ethics, and Human Resources frameworks? So, when conducting this research, we have tended to reach *across* disciplines by incorporating perspectives from our colleagues in Management and Communication, as well as those in Psychology, Personal Relationships, Organizational Behavior, Sociology, and Gender Studies. Although we have always aimed to draw from varied disciplines in our research, we have likely overlooked important pieces of scholarship, due, in part, to the limited interdisciplinary synthesizing work in this area.

## 2. Special Issue

In 2021, after 15 years of researching and teaching the subject, we co-authored a comprehensive review of the extant theory and research on PWRs [2]. We named, claimed, and organized the scholarly work on blended relationships that vary in closeness: workplace friendships, workplace romance, and the colloquial “work spouse.” We conceptualized these unique relationships as *personal workplace relationships*. That review, along with our careers’ worth of research on the topic, motivated this Special Issue. In this Special Issue, we present the work of researchers from diverse backgrounds who share the goal of better understanding workplace relationships. This Special Issue represents important contributions from authors. All articles included in our Special Issue were double-blind peer reviewed by at least two reviewers.

Our Special Issue features empirical studies conducted in Italy, South Korea, China, and the United States of America, with samples comprising employees in various fields, including schoolteachers, salespeople, service industry workers, and university professors, among others. The articles in this Special Issue broadly center around the Communication Processes and Preferences involved in PWRs and the Inherent Ethical Implications of these relationships.

In terms of Communication Processes and Preferences in PWRs, topics, such as disclosure, privacy, and social support, are addressed in four empirical survey studies. Building on her prior research on health disclosures at work [56,57], Westerman and colleagues [58] investigated health disclosures and social support among workplace friends. In addition, LaFrance [59] expanded her research program on communication in sexual relationships [60,61] to explore changes in employees’ beliefs about workplace romance’s organizational value and privacy and how these beliefs relate to contemporary workplace romance advice. Veksler and Boren [62] examined the ties among communicatively restricted organizational stress—a construct they introduced [63,64] —and PWRs and social support among university faculty. Likewise, Landolfi and colleagues [65] studied the links among social support, work-family balance, and employee life satisfaction, further developing their prior research on these topics [66,67]. 

The Ethical Implications Inherent in PWRs are considered in terms of instrumentality and antisocial behavior. Our Special Issue features Duck’s [68] theoretical examination of the contextual factors influencing judgments of PWR appropriateness, incorporating issues he has described for decades [69,70,71]. We also feature Fritz’s [72] theoretical analysis of PWRs from a communication ethics perspective, an expansion of her work on professional civility, virtue, and workplace relationships [73,74,75,76,77].

Instrumentality underlies two empirical studies. It is the focus of Henningsen and Henningsen’s [78] experimental investigation of flirting in task groups, research that extends their prior work on attraction and flirting in the group context [79,80]. Instrumentality is also implicit in Yi et al.’s [81] survey study on interpersonal attention and communication in sales settings, complementing their prior research on sales personnel and organizational performance [82].

In terms of antisocial organizational behavior, Eger et al. [83] delved into the consequences of “organizations as family” [84] by introducing “familial” PWRs. They examined how these types of PWRs may be used to enact organizational violence. Likewise, He and Yun [85] studied how superior–subordinate PWRs may lead to unethical behavior on the part of subordinates, furthering their work on leadership in China [86].

We remain grateful to these authors, whose contributions address PWRs and associated communication and organizational behavior. Though we present a forum here, much remains to be learned about how PWRs “come together and come apart” [87], the communicative processes that facilitate, impede, and complicate PWRs, and the impact PWRs have on organizations, their members, and other stakeholders. Therefore, we conclude with some suggestions for future research.

## 3. Future Research Directions

First, we see great promise in exploring PWRs of various types, namely in mentoring, family businesses, entrepreneurial/start-up ventures, and executive coaching. Mentoring relationships may begin with parties’ assignments to the relationship (formal mentorships) or they may develop organically through workplace interactions (informal mentorships) [88]. Regardless of how mentorships are initiated, they may develop into personal (workplace) relationships [89,90,91]. Given the continuing concerns about sexual harassment in the workplace, informal mentoring in relationships in which sexual orientations align (e.g., a bisexual male and heterosexual female employee, two heterosexual cross-sex employees) is an especially complex type of PWR to unpack [92,93].

Second, we recommend investigating PWRs in the context of family businesses. The dynamics among organizational members characterized by blood relations, marriage, cohabitation, romance, and/or friendships are underexplored, as are non-familial employees’ roles, perceptions, and responses involved in these dynamics. For instance, how are perceptions of organizational justice affected by one’s familial (or non-familial) status? How do former spouses and romantic partners continue to work together after relationship dissolution [10]? What do shareholders do in reaction to businesses marked by changes in familial relationships (e.g., divorce)? The critically acclaimed and popular HBO television series “Succession” testifies to the public appeal of relationships within such contexts.

Third, the personal and professional relationships present in the initiation and success of start-ups, small businesses, and other entrepreneurial activities offer several avenues to be explored in future research. Many small businesses begin with friends or family members collaborating on an idea, financially investing in the venture, and/or helping to launch or manage the business [94]. What happens when the friendship dissolves or the friends disagree on whether to bring a spouse or one’s children into the business? Furthermore, how may the PWRs influence creativity, innovation, the development of ideas, and professionalism [95,96,97], as well as the long-term success of the organization and the personal relationships among the members?

Fourth, we encourage further exploration of formal organization-based relationships that may develop into personal relationships (e.g., friendships) or result from them but do not involve the partners being employed by the same organization. Student–instructor [98], pastor–parishioner [99], and doctor–patient [100] relationships fall into this category. Although these relationships may never develop into PWRs, they are, nonetheless, characterized by the regular crossing of the border between the public and private lives of one or both of the relational partners. For example, students and their professors may socialize outside the classroom (e.g., go to bars together) or engage in romantic relationships. Additionally, church members often disclose intimate and personal details (life domain) to spiritual leaders (their work domain). Future research should continue to examine how these multiple role relationships and border crossings impact the personal and professional domains of the relational partners.

Fifth, an important understudied area involves people who live where they work. Examples of these situations include priests and nuns who live with other priests and nuns, deployed military members living in shared spaces, building superintendents, resident assistants in college dormitories, and various other occupations. In these specific situations, individuals are physically crossing from the work domain into their life domain, which is shared with other members of their work domain. Naturally, discussions of work likely take place at home. Problematically, issues of privacy arise in trying to maintain a personal life separate from a colleague with whom one lives. Future research should examine these unique careers with physically and psychologically blended work and life domains.

Sixth, we recommend the investigation of PWRs within the context of executive coach–client/protégé relationships [101,102]. Executive coach–client/protégé relationships are related to multiple role relationships (e.g., pastor–parishioner) but are distinct from them in that executive coaches are usually contracted by the given organization for a fixed period of time; clients/protégés are full-time, relatively long-term employees in the same organization. In this context, executive coaches and clients/protégés interact in their professional capacities under the auspices of the same organization, yet their goals and relationships to the organization are quite different. How these complex circumstances and the potentially competing motives and goals of the relational partners impact the development, maintenance, and disengagement of coach–client PWRs are prime topics for PWR theory building and research.

Seventh, we recommend the exploration and interrogation of the ways in which organizations attempt to control and use members’ sexuality, emotion, and personal connections for their benefit [103,104]. Excellent work has been conducted in this regard as it pertains to aesthetic labor [105]. We argue that it also occurs in organizational efforts to build strong, close, and supportive teams that willingly work long hours together, all while these same organizations maintain policies that prohibit workplace romance. Furthermore, as scholars [106] and consultants [107] have pointed out, a problematizing approach to studying popular organizational discourses of “employee engagement”, “passion”, “play”, and “loving what you do” is called for. Concepts, such as Eisenberg’s [108] (p. 139) “jamming,” i.e., “instances of fluid behavioral coordination that occur without detailed knowledge of personality”, and Csikszentmihalyi’s [109] “flow,” i.e., an optimal psychological state in which one becomes so involved in an enjoyable activity that nothing else seems to matter, may be particularly relevant to this work.

Finally, we continue to share concerns about an organization’s ability to legislate employees’ personal relationships [2,110]. The implications of the contradictory messages sent by organizations that encourage some types of PWRs (e.g., friendships) but prohibit others (e.g., workplace romances) should be examined. That is, if an organization encourages closeness among employees to serve the organization’s best interest (employee retention, productivity, etc.), how can it also seek to limit the closeness that serves the employees’ personal interests? In this way, organizations are encouraging and restricting employees by saying “get close, but not too close.”

Such PWR policies may also be viewed as offending the dignity and respect to which human beings are entitled. Fiori-Khayat [111] invokes Kantian duty-based ethics in arguing that restrictive PWR policies reject the inherent dignity of employees by treating them only as a means to an (organization’s) end and by denying their ability to engage in rational thought, moral discernment, and responsible behavior. We previously articulated similar concerns [2] and offered recommendations for practice. Note that we do not suggest that organizations have no policy about close relationships; instead, we recognize that close relationships among employees are probable, and organizations should have fair and realistic practices in place that protect the dignity of the relationship partners [111], the rights of third-party employees, and the legal and financial standing of the organization. For instance, companies may wish to consider having a voluntary formal online disclosure process that treats this relationship information as private.

We also recognize that the organizational regulation of employees’ PWRs is not a universal practice. For instance, French law prohibits limiting employees’ rights “to have personal relationships (whether friendly, amorous, sexual, extramarital, or otherwise) with each other” [111] (p. 212). Future research and practice should consider cultural/national differences, both norm-based and legal, that may impact PWR expectations, behaviors, and policies.
